# Cyclohexa-1,4-dienes in transition-metal-free ionic transfer processes

**DOI:** 10.1039/c7sc01657c

**Published:** 2017-05-24

**Authors:** Sebastian Keess, Martin Oestreich

**Affiliations:** a Institut für Chemie , Technische Universität Berlin , Strasse des 17. Juni 115 , 10623 Berlin , Germany . Email: martin.oestreich@tu-berlin.de

## Abstract

Safe- and convenient-to-handle surrogates of hazardous chemicals are always in demand. Recently introduced cyclohexa-1,4-dienes with adequate substitution fulfil this role as El^+^/H^–^ equivalents in B(C_6_F_5_)_3_-catalysed transfer reactions of El–H to π- and σ-donors (C=C/C≡C and C=O/C=N). Surrogates of Si–H/Ge–H, H–H and even C–H bonds have been designed and successfully applied to ionic transfer hydrosilylation/hydrogermylation, hydrogenation and hydro-*tert*-butylation, respectively. These processes and their basic principles are summarised in this Minireview. The similarities and differences between these transfer reactions as well as the challenges associated with these transformations are discussed.

## Abstract 2

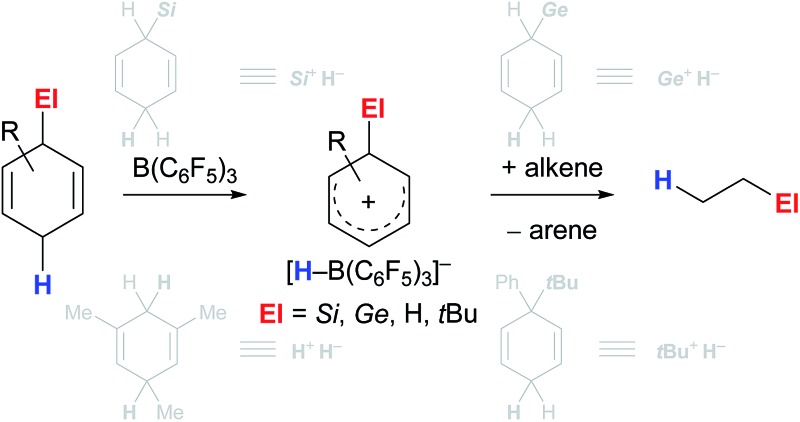
Adequately substituted cyclohexa-1,4-dienes with an electrofuge attached to one of the bisallylic carbon atoms serve as surrogates for small molecules.

## Concept

Transfer processes represent a practical strategy for performing challenging bond formations or avoiding handling hazardous reagents. Limited mainly to transfer hydrogenation^1^ for a long time, this technique has recently emerged as a powerful approach for the application of various toxic, flammable/explosive and/or gaseous chemicals that have otherwise only been rarely used in synthetic chemistry.^2^

**Scheme 1:**
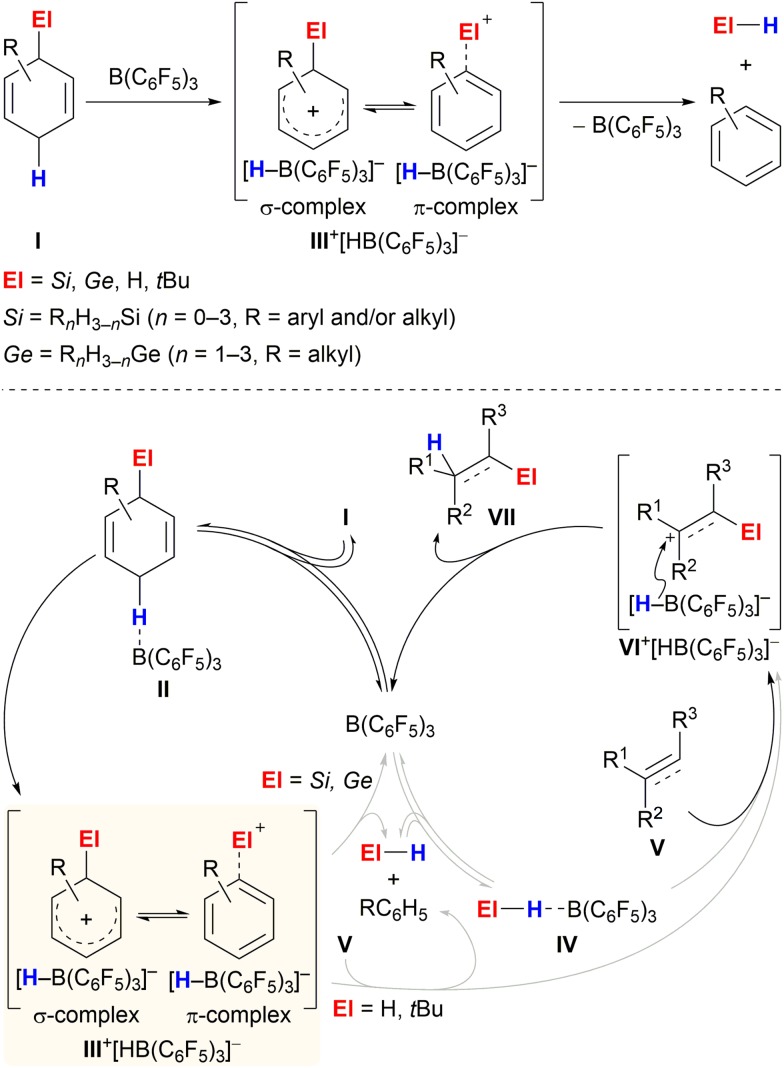
Reaction of cyclohexa-1,4-dienes with B(C_6_F_5_)_3_ in the absence (top) and presence (bottom) of π-basic substrates.

The aptitude of adequately substituted cyclohexa-1,4-dienes **I** to engage in ionic transfer reactions as synthetic equivalents of El^+^/H^–^ (El = Si,^3^ Ge,^4^ H,^5^ *t*Bu^6^) was demonstrated by our laboratory during the last years (Scheme 1). The underlying concept relies on the ability of diene **I** to transiently form ion pair **III** ^+^[HB(C_6_F_5_)_3_]^–^ by B(C_6_F_5_)_3_-mediated hydride abstraction from the bisallylic methylene group (**I** → **III** ^+^)^7^ and subsequently release electrofuge El^+^; aromatisation to furnish the respective arene is exploited as the driving force (Scheme 1, top). The fate of Wheland complex **III** ^+^ was shown to be dependent on the nature of the attached El group, following divergent pathways: El–H release and subsequent activation by B(C_6_F_5_)_3_ or direct delivery of electrofuge El^+^ to substrate **V** (Scheme 1, bottom, grey pathways). Transfer hydrosilylation (El = Si)^3^ or hydrogermylation (El = Ge)^4^ were shown to pass through two interdependent catalytic cycles, liberating the hydrosilane and hydrogermane, respectively (**III** ^+^ → El–H + RC_6_H_5_, left cycle), followed by B(C_6_F_5_)_3_-catalysed El–H bond activation. The thus-formed η^1^-adduct **IV** ^8^ then participates in the reduction of C–C multiple bonds (right cycle).^9,10^ Conversely, transfer hydrogenation^5^ and hydro-*tert*-butylation^6^ proceed by direct transfer of the electrofuge El^+^ from Wheland intermediate **III** ^+^ onto substrate **V** to eventually furnish adduct **VII** after hydride reduction by [HB(C_6_F_5_)_3_]^–^ (**III** ^+^ + **V** → **VI** ^+^ → **VII**).^11^ Consistent with this dichotomy, liberation of the El–H functionality from **I** occurs even in the absence of a Lewis-basic substrate for hydrosilanes/hydrogermanes (El = Si and Ge)^3,4^ while degradation of the H–H and C–H surrogates (El = H and *t*Bu) proceeds only slowly at room temperature.^5,6^

The illustrated concept forms the foundation for all developed transition-metal-free ionic transfer processes using cyclohexa-1,4-dienes **I** as transfer reagents. This Minireview summarises the recent advances in these transformations. It outlines and discusses the challenges and limitations as well as the differences and similarities of the individual transfer processes.

## Transfer reagents

**Fig. 1:**
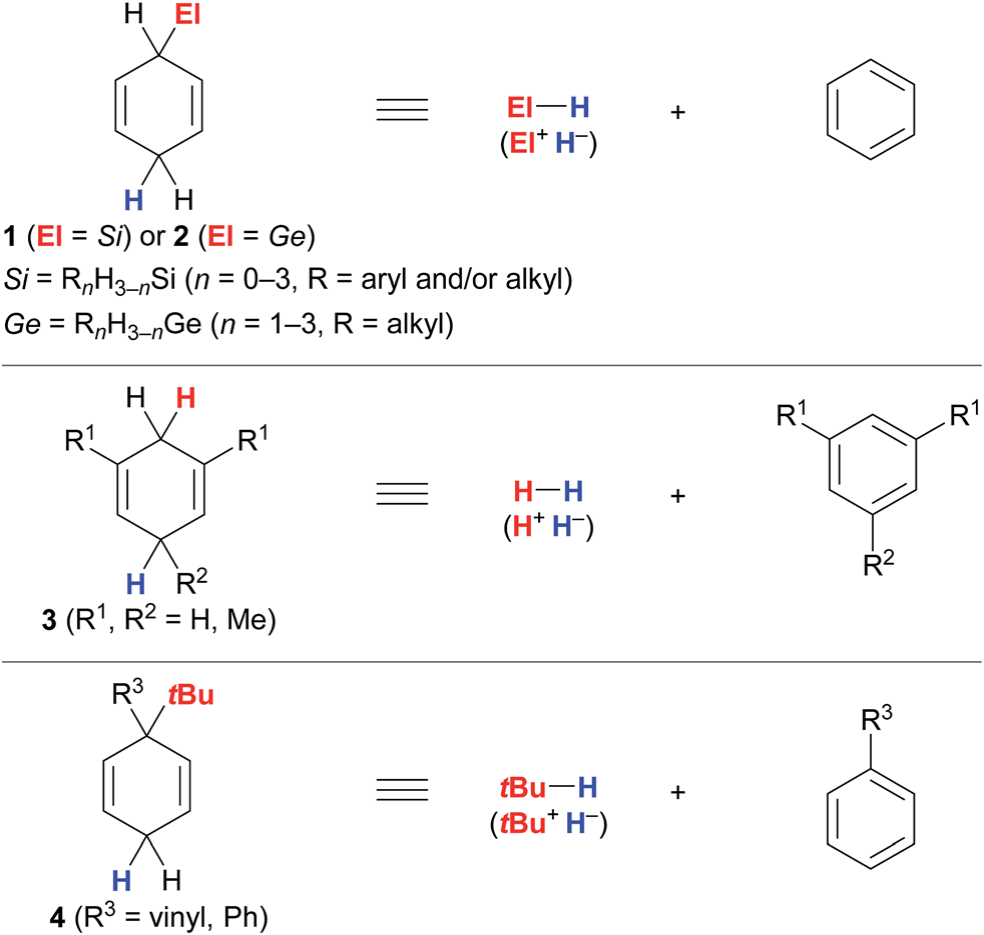
Substituted cyclohexa-1,4-dienes as synthetic equivalents of hydrosilanes/hydrogermanes (top), dihydrogen (middle) and isobutane (bottom).

The successful implementation of cyclohexa-1,4-dienes **I** in the different transfer reactions, *i.e.*, transfer hydrosilylation/hydrogermylation, transfer hydrogenation and transfer hydro-*tert*-butylation, required deliberate modification of the substitution pattern of the cyclohexa-2,5-dien-1-yl unit (Fig. 1).

Unsubstituted cyclohexa-2,5-dien-1-ylsilanes **1** and -germanes **2** cleanly transform into the corresponding hydrosilane or hydrogermane and benzene at room temperature when treated with B(C_6_F_5_)_3_ (Fig. 1, top).^3,4^ Essential for this transformation to proceed is sufficient hydridic character of the bisallylic C(sp^3^)–H bond in **1** due to hyperconjugation with the C(sp^3^)–Si bond and the associated stabilisation of the resulting low-energy Wheland complex **III** ^+^[HB(C_6_F_5_)_3_]^–^ (*cf.* Scheme 1),^12^ as supported by computational studies by Sakata and Fujimoto.^9^ Comparable stabilisation from the C(sp^3^)–Ge bond is expected to facilitate the release of hydrogermanes from surrogates **2**. Conversely, dihydrogen surrogates **3** are devoid of this stabilisation and require electron-donating substituents at C1/C5 (**3a**, R^1^ = Me, R^2^ = H)^5*a*^ or C1/C3/C5 (**3b**, R^1^ = R^2^ = Me)^5*b*^ to lend stabilisation to the resulting high-energy Wheland intermediates **III** ^+^[HB(C_6_F_5_)_3_]^–^ (middle), as well as to suppress undesired reaction pathways, *e.g.*, dihydrogen release or cationic heterodimerisation of reactants. While unsubstituted cyclohexa-1,4-diene (**3c**) favoured side reactions in the transfer hydrogenation of alkenes catalysed by the Lewis acid B(C_6_F_5_)_3_,^5*b*^ Brønsted acids such as Tf_2_NH were shown to selectively mediate transfer hydrogenation from this surrogate.^5c,13^ Likewise, adjustment of the substitution pattern at the cyclohexa-2,5-dien-1-yl core was necessary for the design of the transfer reagents **4** for the B(C_6_F_5_)_3_-catalysed transfer hydro-*tert*-butylation (bottom).^6^ Another substituent “*ipso*” to the *tert*-butyl group in **4** had to be introduced to avoid competing proton release from that position.

## Transfer hydrosilylation/hydrogermylation

**Fig. 2:**
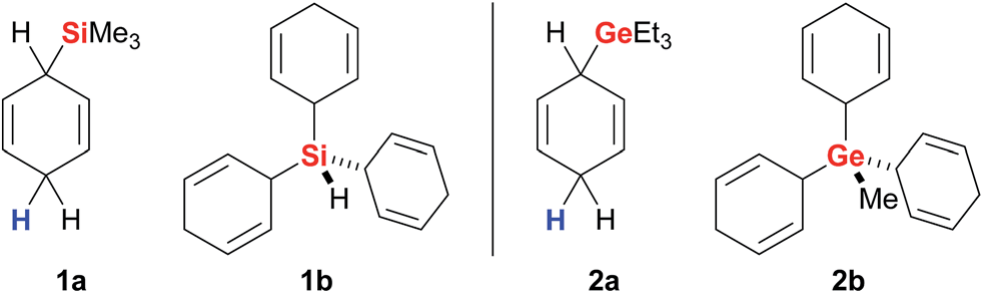
Representative cyclohexa-1,4-dienes as surrogates of hydrosilanes (left) and hydrogermanes (right).

Simonneau and Oestreich introduced cyclohexa-1,4-dienes as reagents in ionic transfer processes^14^ and provided the proof-of-principle for the concept outlined above by employing surrogate **1a** as an equivalent of gaseous Me_3_SiH (Fig. 2, left).^3*b*^ Surrogates of various other hydrosilanes, *e.g.*, functionalised (EtO)_3_SiH,^3*d*^ were prepared and successfully tested in B(C_6_F_5_)_3_-catalysed ionic transfer hydrosilylations (not shown).^3*a*^ The development of solid **1b** as an easy-to-handle surrogate of pyrophoric and explosive SiH_4_ disclosed a rare strategy for the use of monosilane in organic synthesis.^3e,15^ Likewise, related **2a** and **2b** are surrogates of Et_3_GeH and gaseous MeGeH_3_ (Fig. 2, right) that enabled the first examples of transfer hydrogermylation.^4^

**Scheme 2:**
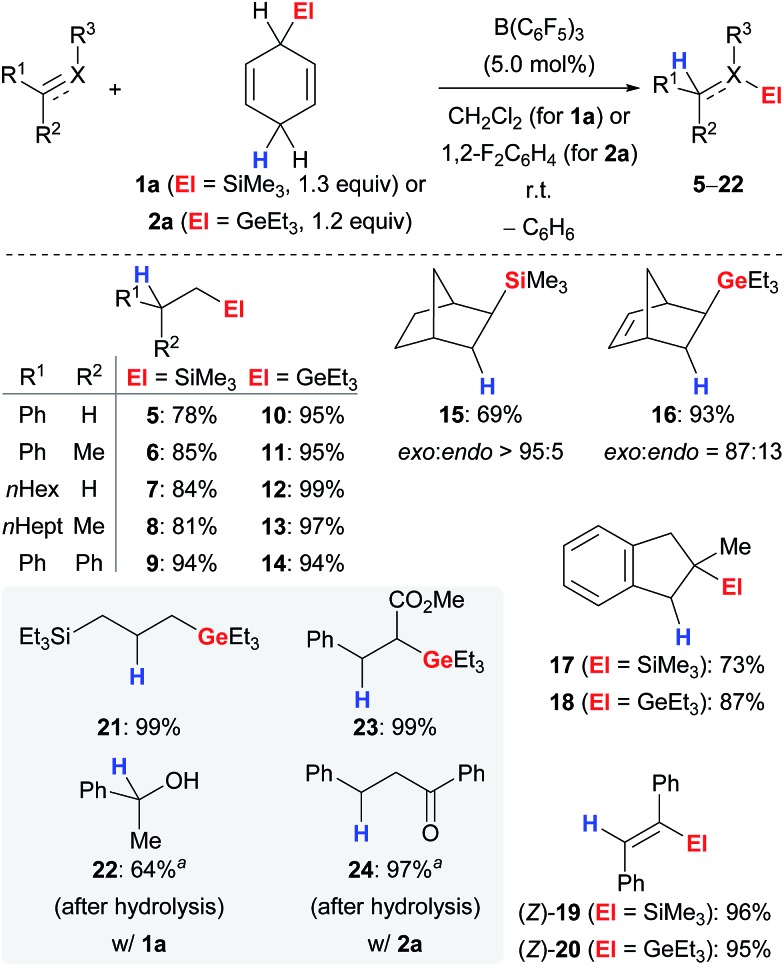
Transfer hydrosilylation and hydrogermylation of C–X multiple bonds using surrogates of Me_3_SiH and Et_3_GeH. ^*a*^Performed at 90 °C.

The ionic transfer hydrosilylation and hydrogermylation of π-basic substrates, *i.e.*, alkenes and alkynes,^16^ proved to be applicable to a wide range of unfunctionalised derivatives (Scheme 2).^3b,d,4^ Both transfer processes proceeded at room temperature using catalytic amounts of the Lewis acid and a slight excess of surrogate **1a** or **2a** in CH_2_Cl_2_ or 1,2-F_2_C_6_H_4_, respectively. Terminal (→**5–14**), *i.e.*, mono- and 1,1-disubstituted, as well as 1,2-disubstituted (→**15–16**) and trisubstituted (→**17–18**) alkenes were compatible with the transfer protocols and furnished tetraorganosilanes and -germanes in high yields. Reduction of an internal electronically unbiased alkyne selectively yielded the product of *trans*-addition (→(*Z*)-**19**–(*Z*)-**20**) whereas transfer hydrogermylation of electronically biased ethyl 3-phenylpropiolate proceeded selectively with *cis* addition, and the ester group was perfectly compatible (not shown).^16*b*^ The *exo* selectivity in the reduction of norbornene (→**15**) and norborna-2,5-diene (→**16**) and predominant *cis* diastereoselectivity in the hydrosilylation of 1-methylcyclohexene (not shown) as well as the absence of products of radical cyclisation in the hydrogermylation of an acyclic 1,6-diene (not shown) confirmed the ionic nature of the mechanism for both transfer reactions.^4,9^ The regioselective formation of **17** and **18** emphasises the favoured formation of a benzylic (secondary) carbocation over a tertiary. A discrepancy in the performance of both surrogates **1a** and **2a** was observed in the reduction of functionalised substrates (grey box). Allyltriethylsilane reacted cleanly in the transfer hydrogermylation (→**21**) whereas only decomposition was observed when subjected to the setup of the transfer hydrosilylation (not shown). Acetophenone yielded alcohol **22** as product of hydrosilylation,^17^ but hydrogermylation of α,β-unsaturated esters and ketones furnished products with untouched carboxyl (→**23**) and carbonyl (→**24**) groups, respectively.

**Fig. 3:**
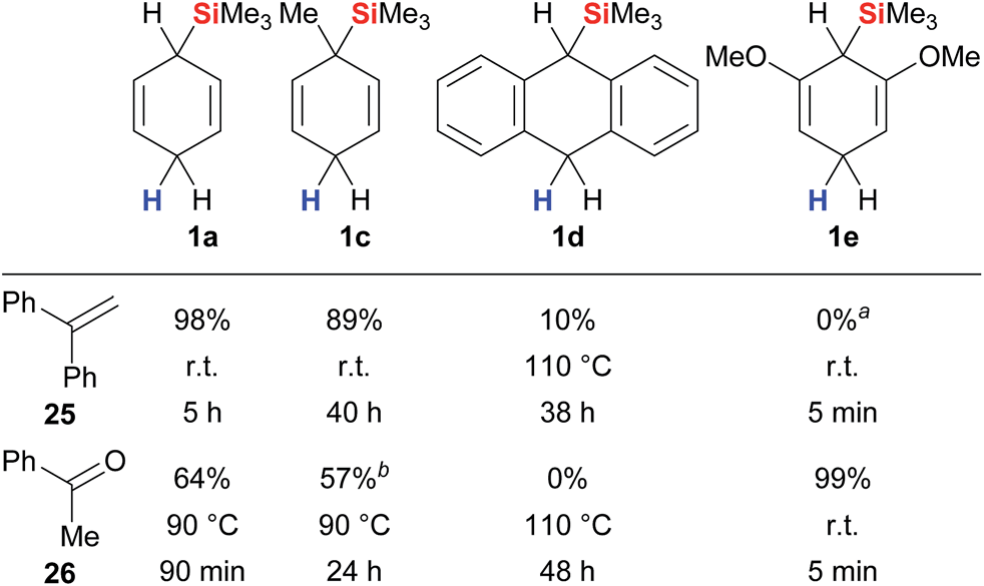
Interplay between surrogate structure and reactivity. ^*a*^Surrogate **1e** fully consumed. ^*b*^Partial deoxygenation to styrene.

A systematic study was recently reported by Oestreich and co-workers that provides comprehensive insight into the parameters that govern the transfer hydrosilylation.^3*d*^ The analysis includes surrogates **1** with modified electronic and steric properties, fully or partially fluorinated triarylboranes as well as representative π- and σ-basic substrates.^18^ Selected data of this study are summarised in Fig. 3. Cyclohexa-1,4-diene **1a** reacts readily with π-donor **25** at room temperature while elevated temperatures are required to split the Lewis acid/base adduct of σ-donor **26** and the borane catalyst (column 1). Derivative **1c** with a methyl group *ipso* to the departing silicon group was less reactive than parent **1a** (column 2), as was surrogate **1d** bearing an extended π system (column 3). Introduction of +M substituents as in **1e** significantly increased the reactivity due to an enhanced hydricity of the bisallylic methylene group, yielding quantitative conversion of σ-basic acetophenone (**26**) at room temperature within minutes (column 4). Conversely, the σ-donating methoxy substituents in resorcinol-derived **1e** outcompete π-basic 1,1-diphenylethylene (**25**) for the transfer of the silicon electrophile, and only cleavage of the ether groups of **1e** was observed (column 4).

**Scheme 3:**
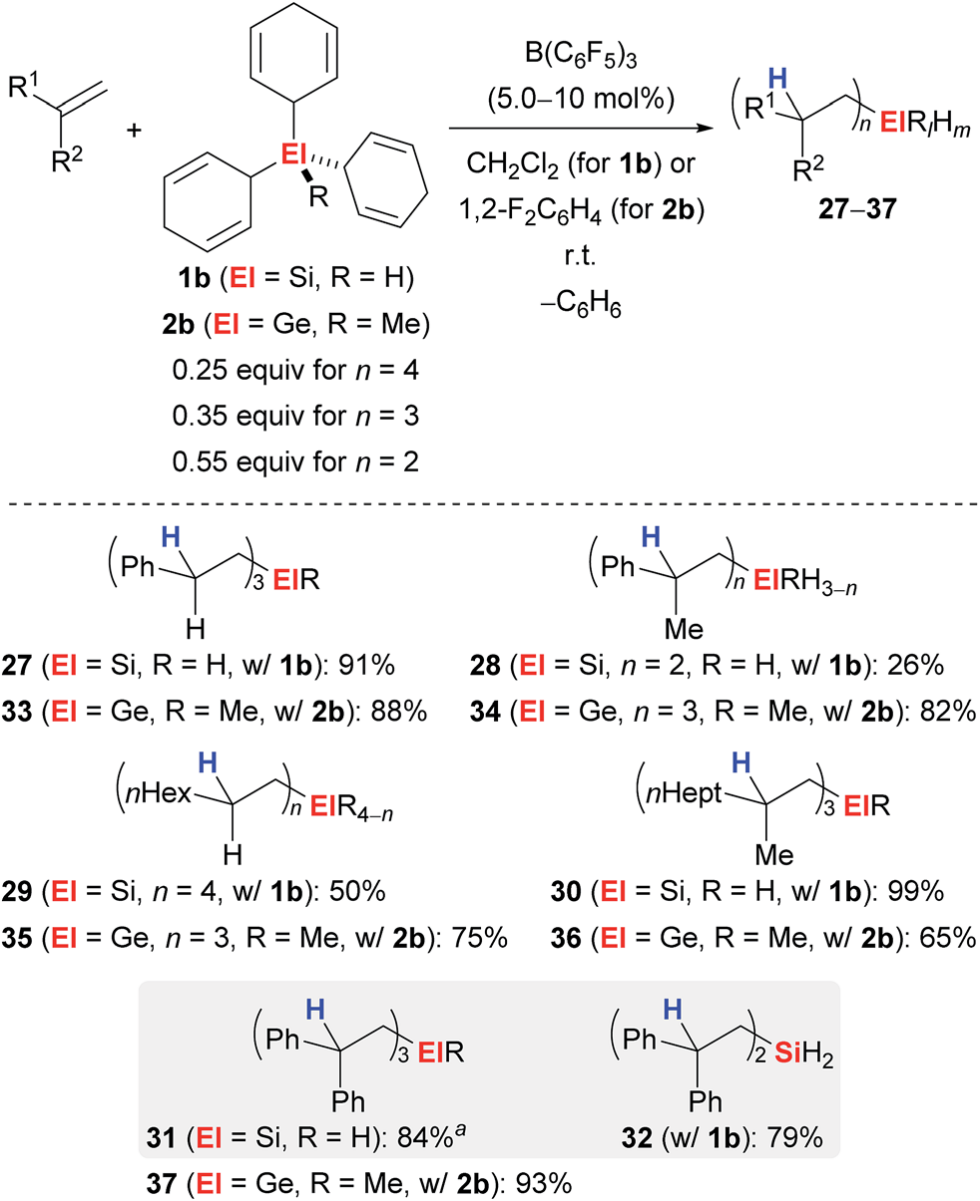
Transfer hydrosilylation/hydrogermylation of alkenes with surrogates of monosilane or methylgermane. ^*a*^Dicyclohexa-2,5-dien-1-ylsilane was used as the surrogate.

Simonneau and Oestreich were able to further advance this strategy by introducing **1b** as a surrogate of SiH_4_, a silane that is rarely used by synthetic chemists due to the associated safety issues.^3*e*^ Later, this approach was unsuccessfully tested to access the related monogermane GeH_4_, and surrogate **2b** as equivalent of MeGeH_3_ was prepared instead.^4^ Both surrogates **1b** and **2b** were shown to liberate SiH_4_ and MeGeH_3_, respectively, upon treatment with catalytic amounts of B(C_6_F_5_)_3_ followed by *n*-fold hydrosilylation or 3-fold hydrogermylation of typical alkenes (Scheme 3). Monohydro- (→**27**,**30**,**31**), dihydro- (→**28**,**32**) and tetraalkyl-substituted silanes (→**29**) became accessible dependent on the steric demand of the alkene; the degree of substitution at the silicon atom can usually not be controlled by the stoichiometry of the reagents. However, for 1,1-diphenylethylene (**25**), reversal of the chemoselectivity, that is the formation of **31** over **32**, was achieved by adjustment of the stoichiometry and the use of di(cyclohexa-2,5-dien-1-yl)silane instead of **1b** (grey box). Also, this method allowed for the mild preparation of tetraalkyl-substituted germanes (→**33–37**).

## Transfer hydrogenation

**Scheme 4:**
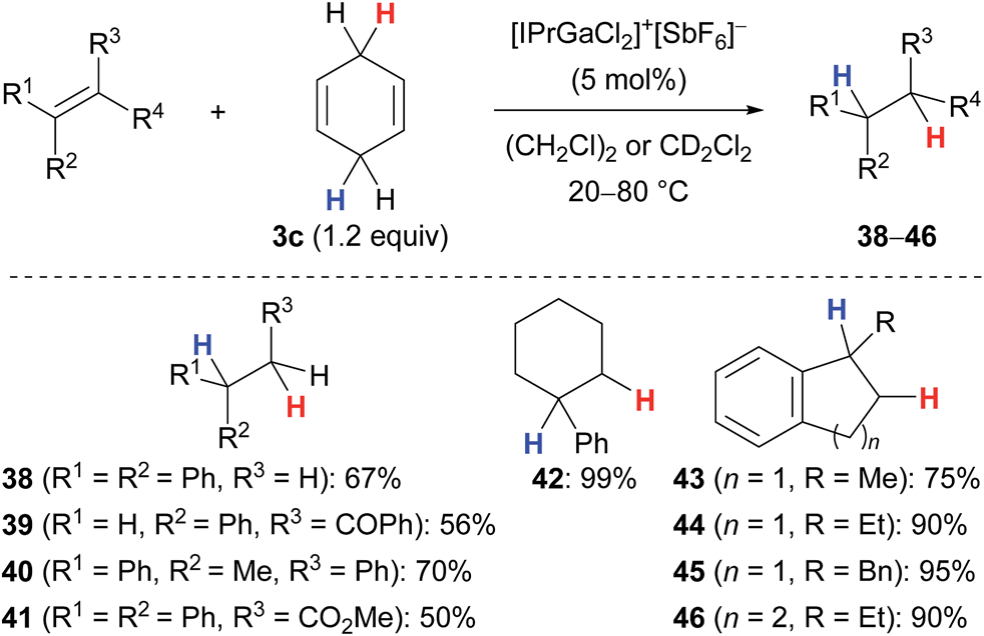
Gallium(iii)-assisted transfer hydrogenation of alkenes.

Kihara and co-workers introduced cyclohexa-1,4-diene (**3c**) as the dihydrogen source in the Lewis acid-catalysed reduction of dithioacetals to the corresponding sulfides (not shown).^19,20^ Later, the research group of Gandon reported a gallium(iii)-assisted transfer hydrogenation of alkenes using the same hydrogen donor **3c** (Scheme 4).^13,21^ Their protocol was applicable to 1,1-disubstituted (→**38**), 1,2-disubstituted (→**39**) as well as trisubstituted acyclic (→**40–41**) and cyclic (→**42–46**) alkenes and tolerated ketone (→**39**) or ester (→**41**) functionalities. Tetrasubstituted alkenes or those without an aryl substituent were unreactive (not shown).

**Scheme 5:**
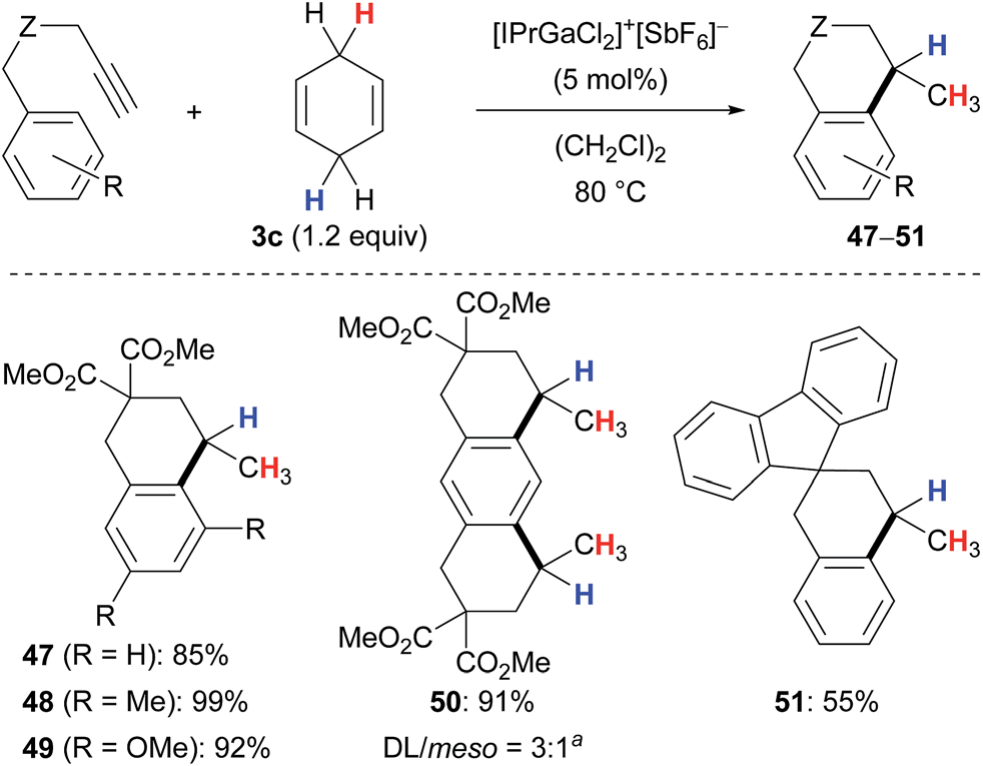
Gallium(iii)-assisted hydrogenative cyclisation of alkynes. ^*a*^2.4 equiv. of **3c** used.

Aryl-substituted alkynes participated in a cascade hydroarylation/transfer hydrogenation sequence catalysed by the same gallium(iii) complex with cyclohexa-1,4-diene (**3c**) as reductant to afford dicyclic (→**47–49**) as well as tricyclic (→**50**) products in high yields (Scheme 5).^13^ The formation of pentacyclic **51** gave a significantly lower yield. Although the mechanism of the gallium(iii)-assisted transfer hydrogenation has not been studied in detail yet, an ionic process was proposed for the dihydrogen transfer (not shown).^13*a*^ Later, it was demonstrated that the transformations depicted in Schemes 4 and 5 work equally well with an indium(iii) complex (not shown).^13*b*^

**Scheme 6:**
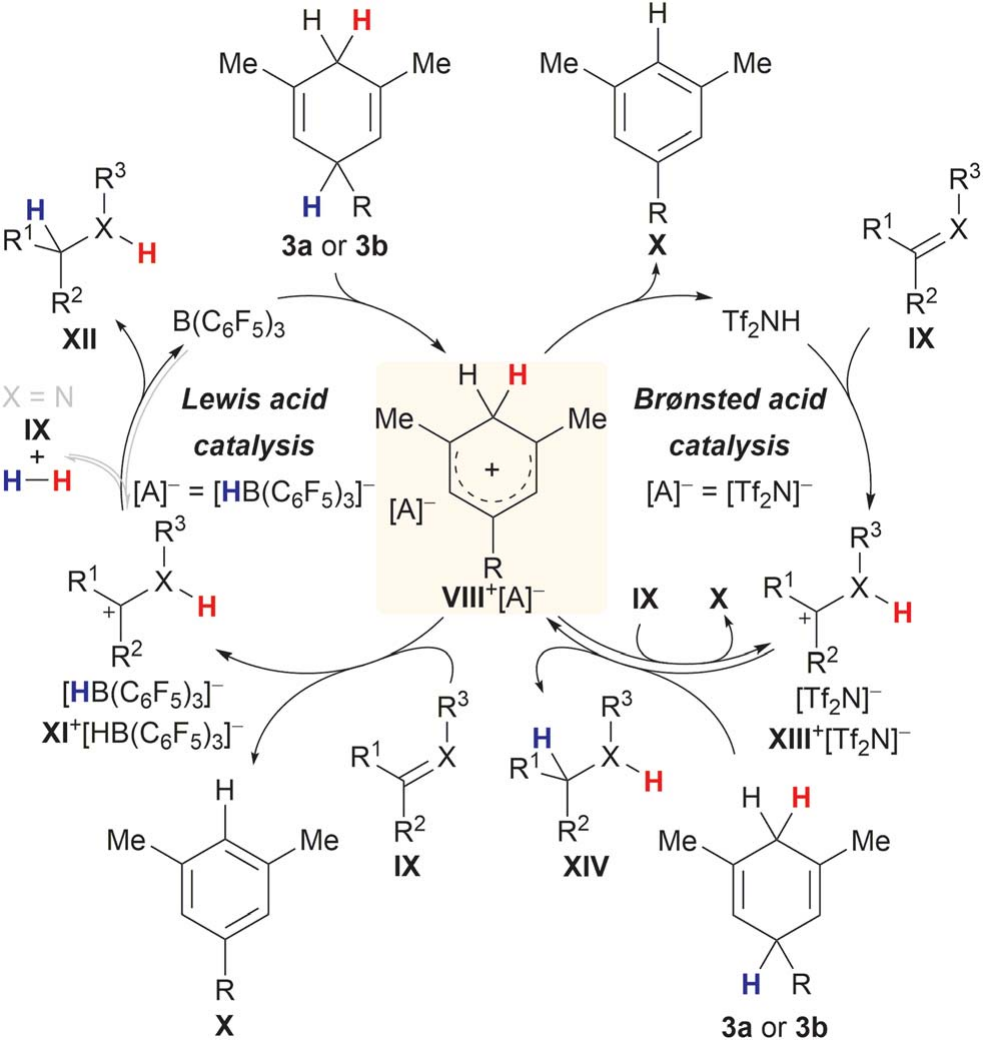
Catalytic cycles for Lewis and Brønsted acid-catalysed transfer hydrogenation of imines (X = NPG) and alkenes (X = CH).

Chatterjee and Oestreich disclosed the B(C_6_F_5_)_3_-catalysed ionic transfer hydrogenation of imines and related heteroarenes employing substituted cyclohexa-1,4-dienes **3a** or **3b** as the dihydrogen source.^5*a*^ Later, Grimme and Oestreich showed that this transfer process also works with alkenes and confirmed the mechanism by quantum-chemical calculations.^5*b*^ The catalytic cycle commences with rate-limiting Lewis acid-mediated hydride abstraction from surrogate **3a** or **3b** to give ion pair **VIII** ^+^[HB(C_6_F_5_)_3_]^–^ in low concentration (Scheme 6, left cycle). High-energy Wheland intermediate **VIII** ^+^ acts as a strong Brønsted acid and protonates σ- or π-basic substrate **IX** to furnish ion pair **XI** ^+^[HB(C_6_F_5_)_3_]^–^ together with stoichiometric arene **X**. Dihydrogen release from **VIII** ^+^ in the presence of a Lewis-basic substrate was excluded. Conversely, subsequent hydride transfer from [HB(C_6_F_5_)_3_]^–^ to the carbenium ion in **XI** ^+^ to regenerate B(C_6_F_5_)_3_ concomitant with the formation of product **XII** was proposed to compete with reversible dihydrogen liberation in the case of imines (grey pathway).^22^ The preference of either pathway depends on the electrophilicity of the carbon atom of the iminium ion intermediate as well as the basicity of the imine nitrogen atom. The formation of highly Brønsted-acidic Wheland intermediate **VIII** ^+^ in the course of the Lewis acid-mediated transfer hydrogenation inspired Chatterjee and Oestreich to investigate potentially competing Brønsted-acid catalysis. As part of these studies, these authors successfully showed that reasonably strong Brønsted acids such as Tf_2_NH are equally able to initiate transfer hydrogenation^23^ from cyclohexa-1,4-dienes **3a** or **3b** by catalytically generating the same Wheland intermediate **VIII** ^+^ (right cycle).^5*c*^ It seems plausible that protonation of substrate **IX** occurs from either Brønsted acids **VIII** ^+^ or Tf_2_NH to furnish intermediate **XIII** ^+^[Tf_2_N]^–^. In the absence of the borohydride [HB(C_6_F_5_)_3_]^–^, cyclohexa-1,4-diene **3a** or **3b** steps in as the hydride donor for the reduction of **XIII** ^+^[HB(C_6_F_5_)_3_]^–^, thereby closing the catalytic cycle.^20*g*^

**Scheme 7:**
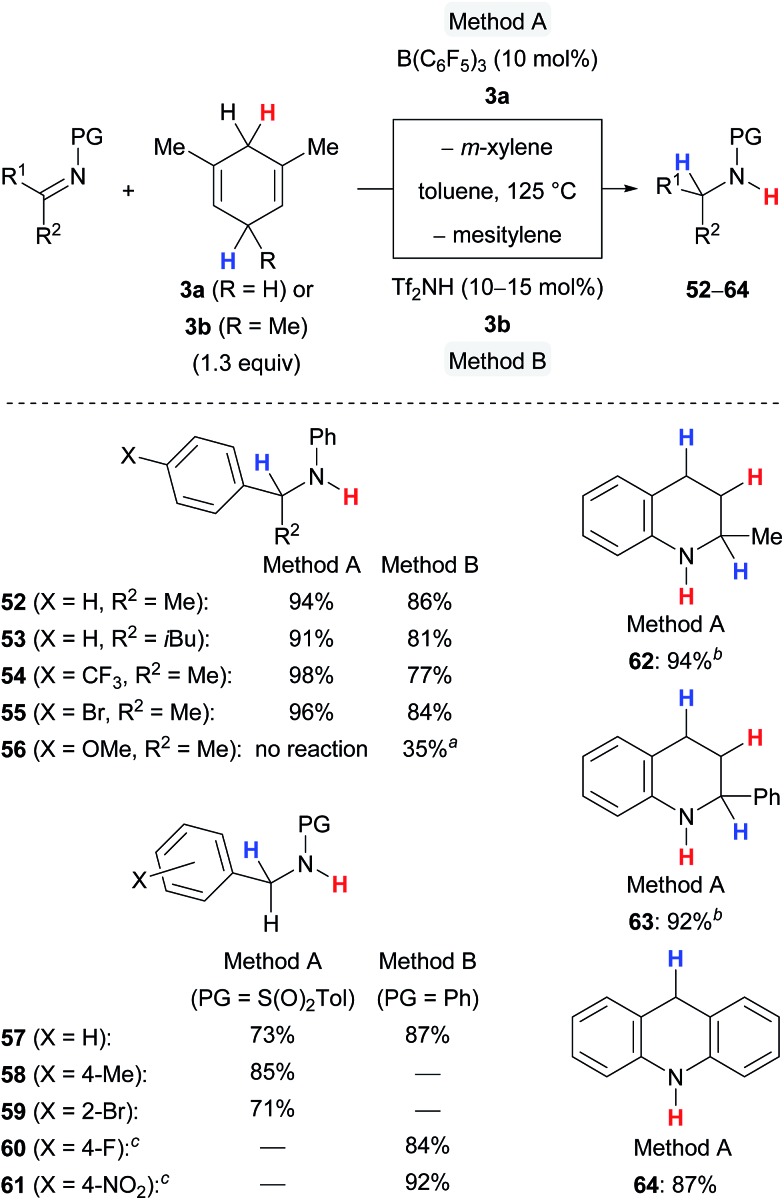
Lewis and Brønsted acid-catalysed transfer hydrogenation of imines and nitrogen-containing heteroarenes. ^*a*^Messy reaction. ^*b*^2.6 equiv. of surrogate **3a** used. ^*c*^Prepared by reductive amination.

The transfer hydrogenation of imines requires forcing reaction conditions, *i.e*., 125 °C and 10 to 15 mol% of the catalyst, and is limited to certain protecting groups at the nitrogen atom to secure optimal steric shielding, sufficient Lewis basicity and stability (Scheme 7).^5a,c^ The protocol is compatible with differently functionalised ketimines (→**52–55**) and aldimines (→**57–61**) and tolerated electron-withdrawing substituents (→**54**,**55**,**59–61**) and even *ortho* substitution (→**59**). A cyclohexanone-derived imine was completely unreactive (not shown), and a 4-anisyl-substituted ketimine showed only moderate reactivity in the presence of the Brønsted acid Tf_2_NH and no reactivity when subjected to catalysis with B(C_6_F_5_)_3_ (→**56**), likely due to lower hydride affinity of the respective iminium ion intermediate. Nitrogen-containing heterocycles participated well in the B(C_6_F_5_)_3_-catalysed transfer hydrogenation affording **62–64** in high yields.

**Scheme 8:**
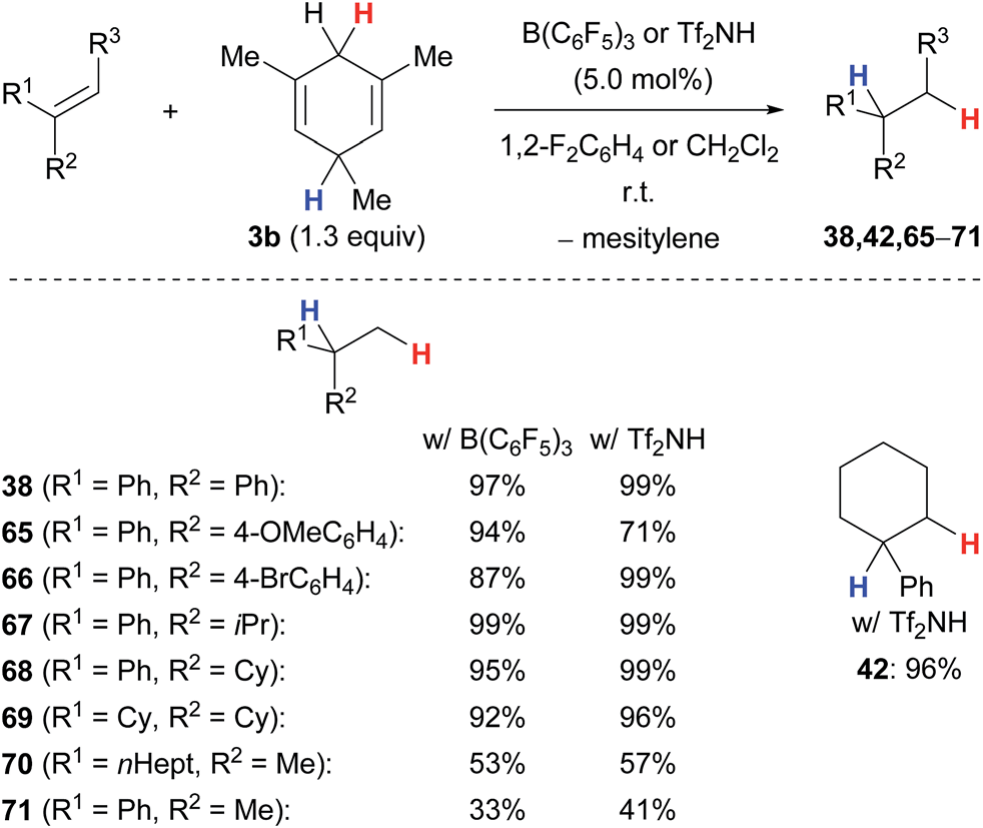
Lewis acid- and Brønsted acid-catalysed transfer hydrogenation of alkenes.

The transfer hydrogenation of π-basic alkenes proceeds equally well with B(C_6_F_5_)_3_ or Tf_2_NH under mild reaction conditions using 5.0 mol% of the catalyst at room temperature (Scheme 8).^5b,c^ The transfer process, however, requires an additional methyl group in the bisallylic position of the cyclohexadienyl group where the hydride abstraction occurs to prevent undesired side reactions, that is liberation of dihydrogen and heterodimerisation of cationic intermediates. The method can be applied to a wide range of 1,1-disubstituted alkenes (→**38**,**65–71**) and works also with trisubstituted derivatives (→**42**). 1,1-Diarylalkenes furnished the corresponding alkanes **38**, **65** and **66** in high yields, irrespective of the electronic properties of the arene. α-Alkyl-substituted styrenes as well as 1,1-dialkylalkenes required sterically demanding substituents, *e.g.*, an isopropyl (→**67**) or a cyclohexyl group (→**68–69**), to prevent thermoneutral cationic dimerisation as observed for **70** and **71**.

## Transfer hydro-*tert*-butylation

Keess and Oestreich introduced 3-*tert*-butyl-substituted cyclohexa-1,4-dienes **4** as transfer reagents in the transfer hydro-*tert*-butylation of alkenes.^6^ This methodology represents an unprecedented approach to install tertiary alkyl groups at carbon frameworks^24^ but competing reaction channels that could not be completely suppressed still limit its synthetic utility.

**Scheme 9:**
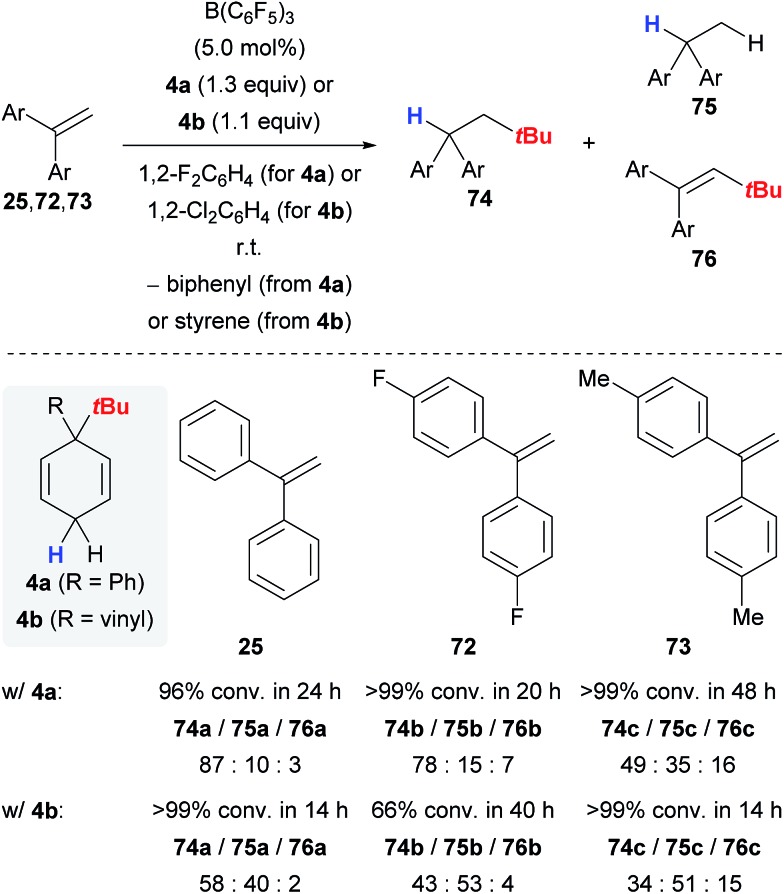
Transfer hydro-*tert*-butylation of 1,1-diarylalkenes.

The transfer of the *tert*-butyl group proceeds smoothly at room temperature with only little excess of transfer reagent **4**, yielding quantitative conversion of the alkene (Scheme 9).^6^ Extensive optimization of the reaction conditions using 1,1-diphenylethylene (**25**) as model substrate could not fully prevent the formation of byproducts **75a** and **76a** (column 1). Electronically modified 1,1-diarylalkenes **72** and **73** were also tested but favoured the formation of the byproducts **75** and **76** to an even greater extent (columns 2 and 3). The influence of the surrogate structure, namely the substituent “*ipso*” to the *tert*-butyl group, is profound, resulting in superior selectivities for surrogate **4a** (R = Ph) compared to **4b** (R = vinyl). In the latter case, separation of the stoichiometrically formed arene byproduct is conveniently achieved as styrene polymerises under the reaction conditions.

**Scheme 10:**
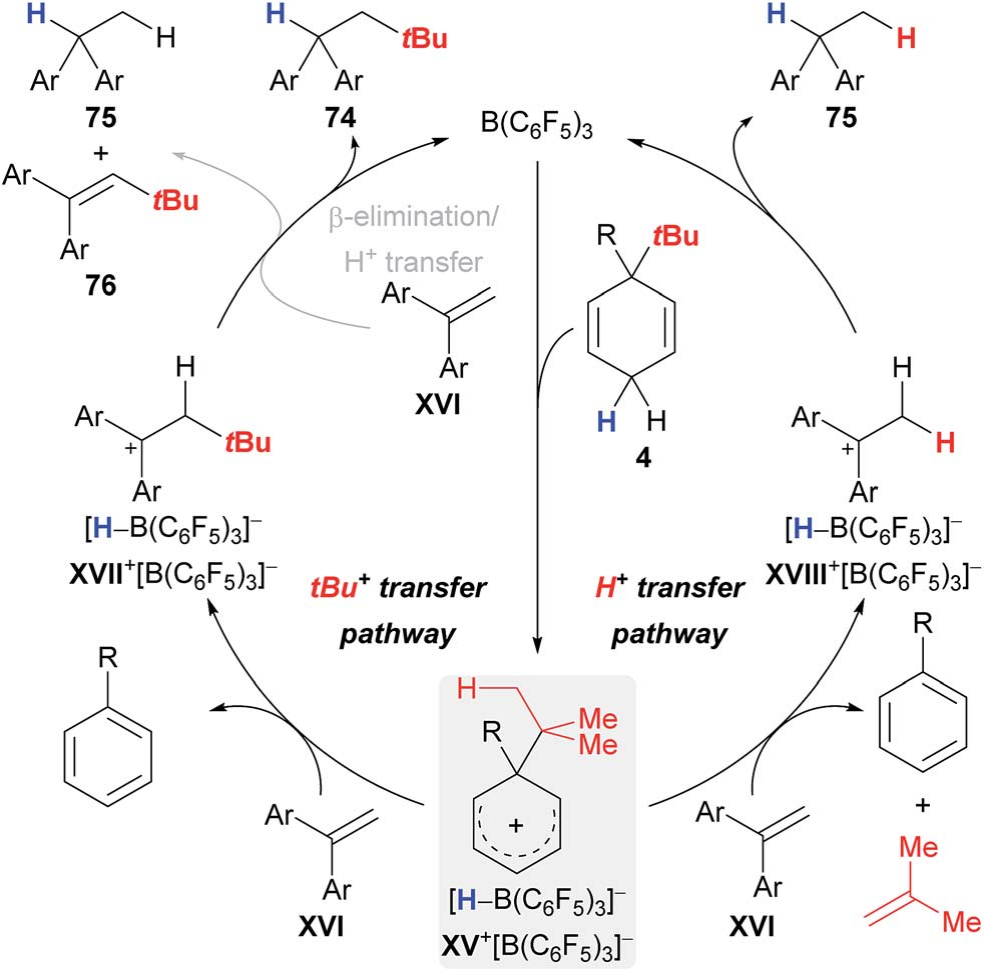
Proposed catalytic cycle for the transfer hydro-*tert*-butylation.

The proposed catalytic cycle that rationalises the pathways for byproduct formation commences with the B(C_6_F_5_)_3_-triggered abstraction of a bisallylic hydride from surrogate **4** to furnish Wheland complex **XV** ^+^[HB(C_6_F_5_)_3_]^–^ (Scheme 10), followed by transfer of either the *tert*-butyl cation (**XV** ^+^ → **XVII** ^+^, left cycle) or a distal proton (**XV** ^+^ → **XVIII** ^+^, right cycle) to alkene **XVI**; stoichiometric liberation of gaseous isobutene likely accounts for the latter pathway. The carbenium ion in **XVIII** ^+^ is eventually reduced by borohydride [HB(C_6_F_5_)_3_]^–^ to afford byproduct **75**, thereby closing the catalytic cycle. Likewise, intermediate **XVII** ^+^ can either directly collapse and form the desired alkane **74** or first transfer a proton from the β position in **XVII** ^+^ to another molecule of alkene **XVI** and form byproducts **75** and **76** after hydride transfer from [HB(C_6_F_5_)_3_]^–^ (grey pathway).

## Outlook

The recent advances in transition-metal-free ionic transfer processes using substituted cyclohexa-1,4-dienes as transfer reagents hint its great promise. While still at the early stages of development, we believe that these transformations are about to emerge as useful synthetic tools. Particularly, unleashing small reactive molecules such as SiH_4_ from cyclohexa-1,4-dienes by straightforward treatment with a Lewis-acid catalyst could also prove valuable for inorganic chemists.

Transfer hydrosilylation is feasible for several π- and σ-donors with a variety of hydrosilane surrogates, particularly of SiH_4_ and (EtO)_3_SiH. Lack of chemoselectivity and, hence, functional-group tolerance is the obvious limitation of this method. That problem is less pronounced in the related transfer hydrogermylation. Issues in the transfer hydrogenation such as hetero- and homodimerisation of the reactants have been successfully addressed by judicious choice of the substituents at cyclohexa-1,4-diene core. The substrate scope for both C=N and C=C bonds is however still relatively narrow. The transfer of a *tert*-butyl group is currently the biggest challenge. While it represents promising precedence for the transfer of carbon electrofuges, the surrogate synthesis still remains unsolved. The design of new (short) synthetic routes and extension to other stabilised carbenium ions as departing groups will hopefully allow for more efficient transfer hydroalkylation reactions in the future.

On the basis of the knowledge gained from these efforts, we will continue improving the existing procedures and devise new El^+^/H^–^ equivalents. We also hope that our findings serve as an inspiration for others in the field.
